# Pain-related Somato Sensory Evoked Potentials: a potential new tool to improve the prognostic prediction of coma after cardiac arrest

**DOI:** 10.1186/s13054-015-1119-y

**Published:** 2015-11-17

**Authors:** Paolo Zanatta, Federico Linassi, Anna Paola Mazzarolo, Maria Aricò, Enrico Bosco, Matteo Bendini, Carlo Sorbara, Carlo Ori, Michele Carron, Bruno Scarpa

**Affiliations:** Department of Anaesthesia and Intensive Care, Intraoperative and Critical Care Neurophysiology in Cardiac Surgery, Treviso Regional Hospital, Azienda Ospedaliera Ulss 9, Piazzale Ospedale 1, 31100 Treviso, Italy; Neuromonitoring Project, Department of Anesthesia and Intensive Care, Treviso Regional Hospital, Piazzale Ospedale, 1, 31100 Treviso, TV Italy; Unit of Neuroradiology, Treviso Regional Hospital, Piazzale Ospedale, 1, 31100 Treviso, TV Italy; Department of Anesthesia and Intensive Care, Padova University Hospital, Via 8 Febbraio 1848, 2, 35122 Padova, PD Italy; Department of Statistical Sciences, Padova University, Via 8 Febbraio 1848, 2, 35122 Padova, PD Italy

## Abstract

**Introduction:**

Early prediction of a good outcome in comatose patients after cardiac arrest still remains an unsolved problem. The main aim of the present study was to examine the accuracy of middle-latency SSEP triggered by a painful electrical stimulation on median nerves to predict a favorable outcome.

**Methods:**

No- and low-flow times, pupillary reflex, Glasgow motor score and biochemical data were evaluated at ICU admission. The following were considered within 72 h of cardiac arrest: highest creatinine value, hyperthermia occurrence, EEG, SSEP at low- (10 mA) and high-intensity (50 mA) stimulation, and blood pressure reactivity to 50 mA. Intensive care treatments were also considered. Data were compared to survival, consciousness recovery and 6-month CPC (Cerebral Performance Category).

**Results:**

Pupillary reflex and EEG were statistically significant in predicting survival; the absence of blood pressure reactivity seems to predict brain death within 7 days of cardiac arrest. Middle- and short-latency SSEP were statistically significant in predicting consciousness recovery, and middle-latency SSEP was statistically significant in predicting 6-month CPC outcome. The prognostic capability of 50 mA middle-latency-SSEP was demonstrated to occur earlier than that of EEG reactivity.

**Conclusions:**

Neurophysiological evaluation constitutes the key to early information about the neurological prognostication of postanoxic coma. In particular, the presence of 50 mA middle-latency SSEP seems to be an early and reliable predictor of good neurological outcome, and its absence constitutes a marker of poor prognosis. Moreover, the absence 50 mA blood pressure reactivity seems to identify patients evolving towards the brain death.

## Introduction

Several efforts have been recently made to improve prognostication in comatose patients after cardiac arrest (CA). While the introduction of targeted temperature management (TTM) issues new challenges in interpreting clinical and neurophysiological findings [1–5], early prediction of a good outcome still remains an unsolved question. A multimodal approach combining clinical, biochemical, neurophysiological, and neuroimaging parameters was proposed to overcome the prognostic failure induced by TTM [6], but it strengthened the accuracy only in predicting a poor outcome. Conversely, recent trials suggested the detection of continuous, reactive, spontaneous brain cortical activity to predict a good outcome [6–8], but unfortunately electroencephalogram (EEG) findings can suffer both from false positives and false negatives, reflecting the dynamism of acute brain damage, which can evolve toward the recovery from brain ischemia or toward worsening damage. False positive EEG finding (i.e., postanoxic status epilepticus in a patient who then recovers) are of critical value for the prognostication because they may discourage physicians from intensifying treatments [9, 10]. Literature shows that in the acute phase after CA, the brain ischemia associated with hypothermia and pharmacological sedation may suppress reactivity and the cortical synaptic background transmission at different levels, hiding the existence of still-viable brain tissue but not functioning in a way that is typical of the ischemic penumbra [11–15].

How can the diagnostic tools in the early phase of postanoxic coma be improved? Neurophysiologists and neurocritical care physicians have recently shown interest in middle-latency somato sensory evoked potentials (ML-SSEP), represented by cortical potentials in the range of 40–70 ms, because they reflect higher-order brain processes represented by cortical-cortical synaptic functions [16–19]. These synaptic functions seem to be required for recovery of consciousness and a good outcome [20–23]. It has also been attested that ML-SSEPs are more sensitive than short-latency SSEP (SL-SSEP, commonly named N20/P25) to the ischemic injury, and their preservation reflects a lower level of brain damage [24–26].

Furthermore, past studies showed that 40–70 ms potentials are localized in the para-Sylvian cortex near the lateral sulcus by the firing of the secondary somatosensory area (SII) and insula [27], that they can be activated by A delta nerve fibers [28, 29], and that they are involved in the cortical representation of pain [30]. Several years later we had the opportunity to replicate the causal effect of A delta conduction fiber on ML-SSEP generation; indeed, we suppressed 40–70 ms potentials triggered by painful stimulus at 50 mA by blocking the median nerve sulcus with local anesthesia [11]. Moreover, we found that 50 mA ML-SSEP stimulation on the median nerves in anesthetized patients is associated with an increase in blood pressure, suggesting an autonomic response to stress [11, 31]. In a preliminary report on a small cohort of postanoxic comatose patients, we showed that ML-SSEP and blood pressure (BP) reactivity triggered by painful electrical stimulations on the median nerves seem to predict, respectively, a good neurological outcome and survival [31]. Finally we attested that the same stimulation paradigm applied to functional magnetic resonance imaging (fMRI) can show the brain network involved in the detection of pain, also called the “pain matrix”: SII, insula, and anterior cingulate cortex [32]. Prognostically, the presence of this neurophysiological cortical activity (and the respective neuroanatomical activity) demonstrates the functional integrity of a network that is multisensory, non-nociceptive-specific, and involved in the processing of salient sensory input related to motor preparation and emotional expression [33, 34]. In other words, in postanoxic coma, the persistence of ML-SSEPs after SL-SSEP reflects the functional integrity of connections that are involved in more complex processes than those of the primary somatosensory cortex (which are detected by SL-SSEPs). On the other hand, the absence of ML-SSEPs after SL-SSEP means that those areas involved in consciousness recovery lack functionality, thus predicting an evolution toward the minimally conscious state [32].

The target of this study is to compare ML-SSEP and BP reactivity triggered by high-intensity stimulation on the median nerves to all the most important clinical, biochemical and neurophysiological parameters that literature attests as early predictors of survival, consciousness recovery and good neurological outcome (i.e., awakening and Glasgow-Pittsburgh cerebral performance categories (CPC) 1–2).

## Methods

### Subjects

From July 2010 to August 2014, 167 postanoxic patients were admitted to ICUs at Treviso Regional Hospital (103 in the general ICU and 64 in the cardiac surgery ICU). As the major inclusion criterion for the present study was the availability of the EEG and SSEP within 72 h of CA, only 46 patients were retrospectively considered. All the neurophysiological evaluations were performed by the same neurophysiologist. None of these patients had previous neurological disease or sepsis. No distinction in recruitment was made for the place (in-hospital or out of hospital) or cause (cardiac or non-cardiac) of CA. The Lund University CA system (LUCAS) was used as a bridge for extracorporeal membrane oxygenation (ECMO) in patients not achieving the return of spontaneous circulation (ROSC) with conventional cardiopulmonary resuscitation (CPR). After ICU admission, patients were evaluated and subjected to TTM with Arctic Sun© 5000 (Medivance, Inc. 321 South Taylor Ave. Suite 200, Louisville, CO 80027), according to protocols described in the literature [35].

### Clinical and biochemical parameters

Time from CA to ROSC was divided into no-flow (time from CA to CPR) and low-flow (time from CPR to ROSC or to the start of ECMO). The initial CA rhythm was categorized into ventricular tachycardia (VT), ventricular fibrillation (VF), pulseless electrical activity (PEA) and asystole (AS). Pupillary reflex (PR) and the Glasgow scale motor score (M) were measured by the intensive care physician at ICU admission. First values of partial pressure of arterial oxygen (PaO_2_), partial pressure of arterial carbon dioxide (PaCO_2_), HCO_3_^−^, pH, glucose and lactates at hospital admission were considered.

The occurrence of hyperthermia (tympanic temperature >37.8 °C) and the highest value of creatinine within 72 h of CA were also reported. Patients requiring pharmacological cardiovascular support were categorized according to three levels: low dosage (dopamine or dobutamine ≤4 μg·kg^−1^·min^−1^, noradrenaline (norepinephrine) ≤0.1 μg·kg^−1^·min^−1^, or adrenaline (epinephrine) ≤0.05 μg·kg^−1^·min^−1^), medium dosage (dopamine or dobutamine >4^−1^ and <8 μg·kg^−1^·min^−1^, noradrenaline >0.1 μg·kg^−1^·min^−1^ and <0.2 μg·kg^−1^·min^−1^, or adrenaline >0.05 μg·kg^−1^·min^−1^ and <0.1 μg·kg^−1^·min^−1^) and high dosage (dopamine or dobutamine ≥8 μg·kg^−1^·min^−1^, noradrenaline ≥0.2 μg·kg^−1^·min^−1^, or adrenaline ≥0.1 μg·kg^−1^·min^−1^).

### Neurophysiological evaluation

Neurophysiological recordings were performed within 72 h after CA (at a mean of 33 ± 22 h) and consisted of EEG (background pattern and reactivity) and SSEP recorded in the same session. SSEP was performed by bilateral stimulation of the median nerves at 3.3 Hz using needle electrodes on both wrists. We considered two steps of electrical stimulation - low intensity (10 mA) and high intensity (50 mA) - to trigger the possible appearance of unilateral or bilateral cortical ML-SSEP. SL-SSEP was defined as present if the cortical N20/P25 response was present on one or both sides. SL-SSEP was defined as bilaterally absent if no reproducible potentials could be identified on either side at a maximum gain of 1 uV per division in the presence of the brachial plexus potential. ML-SSEP was considered present if any reproducible potentials were detected on one or both sides in the range of 30–90 ms and with amplitude over 0.5 uV. Both SL and ML-SSEP were considered in the same recording window of 100 ms. The high-intensity stimulation was also used to trigger EEG and BP reactivity. BP reactivity was considered significant if there was an increase of 10 % over baseline values recorded at 10 mA stimulation. Muscle-paralyzing medication (cisatracurium, 0.15 mg·kg^−1^) was always used during the neurophysiological evaluation to reduce the noise induced by possible muscle artifacts. The ground electrode was placed on the left shoulder for both EEG and SSEP recordings. The impedance was kept below 1 kΩ. EEG and SSEP were recorded using the NIM-Eclipse^©^ Nerve Monitoring System (Medtronic Xomed, Jacksonville, FL, USA).

### EEG recording parameters and classification

Eight bipolar EEG channels were recorded with needle electrodes placed at the standard scalp sites (F3/F4-Cz, C3’/C4’-Cz, T3/T4-Cz, P3/P4-Cz); in particular, C3’ and C4’ were placed 2 cm posterior to C3 and C4, according to the 10/20 international system. The EEG recording parameters were 1 and 40 Hz for low- and high-frequency filters, respectively, with a sampling rate of 250 Hz and hardware bandwidth of 1–100 Hz. EEG patterns were categorized by simplifying Cloostermans’ classification [7] into three grades: grade 1, continuous pattern (diffuse slowing, normal); grade 2, epileptiform pattern; and grade 3, discontinuous pattern (isoelectric, burst suppression, low voltage). We chose this simplified EEG classification for statistical reasons and also because, in our opinion, it is easily interpreted by intensivists. EEG reactivity was considered if background patterns showed any reproducible change (acceleration or slowing, including amplitude variation) upon stimulation at 50 mA.

### SSEP recording parameters

Four bilateral SSEP channels were recorded within a time base of 100 ms: C4’/C3’–Fpz detected the SL (N20/P25)-SSEP and the ML-SSEP; C4’/C3’–right Erb’s point/left Erb’s point detected the interpeak between P14/N18 subcortical potential; Cv–Fpz detected the N13 cervical potential; and right Erb’s point/left Erb’s point–Fpz detected the N9 brachial plexus potential. SSEP was double-filtered at 30 Hz and 500 Hz, respectively for low and high frequency filter. Each trace averaged 100 sweeps; a minimum of three traces were taken during every step. Each step of stimulation lasted 90 s after rejection of trials due to possible artifacts. The stimulus duration was lengthened from 200 μs in the first series of 17 patients [29] to 1000 μs in the remaining patients to increase the possible activation of the Aδ nerve fibers [36, 37].

### Outcome evaluation

Recovery of consciousness was defined as the ability to repeatedly carry out simple commands to more than one physician. CPC outcome evaluation was performed at 6 months after a medical and neurological examination; in the same session, patients were subjected to a neuropsychological evaluation, the results of which were not considered for the following study. Outcome was classified as good (CPC 1–2, corresponding to no or moderate neurological disability), and poor (CPC 3–4–5, corresponding to a severe disability, coma and death).

### Decision to treat

Status epilepticus with normal SL-SSEP was treated only after the neurophysiological evaluation, using incremental doses of midazolam (0.03–0.2 mg·kg^−1^·h^−1^) with or without propofol (3–10 mg·kg^−1^·h^−1^); the most resistant patients were additionally treated with thiopental to achieve the burst suppression pattern for 48 h. Levetiracetam (1 g twice daily) was also used to replace continuous sedation; additional antiepileptic medications were given according to EEG patterns. In patients with bilaterally absent cortical SSEP, malignant unreactive EEG patterns and absent brainstem reflexes, the decision to withdraw supportive care was taken at a physician’s discretion. Patients who persisted in a comatose state for more than 15 days despite the presence of SL-SSEP or SL/ML-SSEP were subjected to brain fMRI with the same stimulation paradigm of the pain-related SSEP to endorse the evoked potential evaluation.

### Ethics

The use of pain is a paradox in the medical practice; while, on one hand, it must be treated to reduce suffering and stress response, on the other hand, it is widely used to explore consciousness in comatose patients. Indeed, the intensivists use the Glasgow coma scale (GCS) daily to investigate consciousness status [38]. Since 2010, our institution has replaced the use of GCS with pain-related (50 mA) SSEP in postanoxic coma, as compared to the GCS, SSEP requires less frequent painful stimulations (at most two stimulations with a 180-s duration) in the first 72 h and represents a more sensitive method to explore brain function (brain somatosensory afferent pathway reactivity versus the efferent motor response of the GCS, which is often blunted by sedation) [31]. As pain is considered an unpleasant experience that involves the conscious awareness of noxious sensations [39], the painful somatic (GCS) or neurophatic (pain-related SSEP) stimulations seem to be ineffective during deep coma, sedation and concomitant global disorders of the EEG signals; none of the patients experience a conscious awareness of noxious sensations. Moreover, in some circumstances, the pain-related ML-SSEP associated with severe EEG disorders can induce the physician to continue therapies instead of abandoning them. Relatives of the patients were always made aware of the use of this additional technique. All procedures performed in this study was in accordance with the ethical standards of the institutional and/or national research committee and with the 1964 Helsinki declaration and its later amendments or comparable ethical standards. For this retrospective type of study formal consent is not required. The manuscript was approved by the Provincial Ethics Committee of Treviso (N. 11; record 852/14).

### Statistical analysis

All recorded parameters were related to three outcome categories (survival, recovery of consciousness, and CPC at 6 months after CA) and divided into two outcome levels (good outcome (CPC 1–2) and poor outcome (CPC 3–5)). To describe the general characteristics of the study population, absolute and relative frequencies were calculated for qualitative data, and mean and standard deviation were calculated for quantitative values (Table 1). To estimate which variables are related to mortality, recovery of consciousness, or 6-month CPC outcomes, Pearson’s chi-square test was calculated for qualitative data and the Mann–Whitney test was calculated for quantitative data. To estimate whether TTM and sedation were associated with EEG reactivity, SL-SSEP and ML-SSEP, or BP reactivity, Student’s *t* test was performed. The Wilcoxon signed-rank test was performed to evaluate whether there were significant differences between 50 mA ML-SSEP, EEG reactivity appearance time, and time of best motor response among patients who had 10 or 50 mA ML-SSEP or EEG reactivity. To investigate whether the presence of unilateral or bilateral ML-SSEP is associated with CPC, Pearson’s Chi-square test was calculated. Relative risk (RR) was calculated to investigate the efficacy of TTM, intra-aortic balloon pumps (IABPs), ECMO, continuous veno-venous hemofiltration (CVVH), coronarography, and inotropic drugs; among out-of-hospital (OHCA) patients, the same test was used to determine if there were significant outcome differences for patients hospitalized in the general and cardiac surgery ICUs. Given the large number of comparisons conducted on the same data and the large number of variables analyzed, we performed the Holm [40] correction for multiple comparisons, which is less conservative than the more frequently used Bonferroni correction. A *p* value <0.05 was considered to be statistically significant. To investigate which variables added significant predictive capacity, for each output variable, we fitted a logistic regression model by selecting relevant variables through a cross-validated stepwise selection procedure. Given the small dataset and the high co-linearity of many variables, we estimated the logistic regression parameters using a bias-reduction maximum-likelihood method [41, 42]. Leave-one-out cross-validation was implemented at each step of the forward selection procedure to choose the best set of predictors for each output variable. Statistical analysis was performed with IBM SPSS Statistics for Windows, Version 19.0 (IBM Corp., Armonk, NY, USA) and with R [43].

**Table 1:** General characteristics of the population

Characteristic	Arithmetic mean	Value standard deviation	Minimum–Maximum
Age (years)	59.37	14.43	20–89
Time no-flow (minutes)	7.67	26.03	4–110
Partial pressure of arterial O_2_ (mmHg)	138.86	112.44	25.8–539
Partial pressure of arterial CO_2_ (mmHg)	48.92	12.91	21.1–81.1
HCO_3_^-^ (mmol/L)	14.85	4.75	7–27.4
Ph	7.13	0.14	6.87–7.41
Glycemia (mg/dL)	307.15	97.84	114–627
Lactates (mmol/L)	9.87	4.05	2.4–20
Highest creatinine during 72 h (mg/dL)	1.84	1.12	0.64–4.8
Characteristic	Absolute frequency Number	Value relative frequency %	CI 95 %
Ventricular fibrillation rhythm of cardiac arrest	32	69.57	55.20–80.92
Pulseless electrical activity rhythm of cardiac arrest	8	17.39	9.08–30.72
Asystole rhythm of cardiac arrest	6	13.04	6.12–25.66
Cardiac arrest due to cardiac cause	32	69.57	55.20–80.92
Cardiac arrest due to non-cardiac cause	14	30.43	19.08–44.80
Patients with out-of-hospital cardiac arrest	35	76.09	62.07–86.09
Patients with in-hospital cardiac arrest	11	23.91	13.91–37.93
LUCAS™ patients	11	23.91	13.91–37.93
Not LUCAS™ patients	35	76.09	62.07–86.09
General ICU patients	18	39.13	26.39–53.54
Heart surgery ICU patients	28	60.87	46.46–73.61
Low-dosage patient’s intravascular drug support	10	21.74	12.26–35.57
Medium-dosage patient’s intravascular drug support	9	19.57	10.65–33.18
High-dosage patient’s intravascular drug support	27	58.70	44.34–71.72
Patients with intra-aortic balloon pump	16	34.78	22.68–49.23
Patients with no intra-aortic balloon pump	30	65.22	50.77–77.32
Patients who had coronary angiography	28	60.87	46.46–73.61
Patients who did not have coronary angiography	18	39.13	26.39–53.54
Percutaneous transluminal coronary angioplasty/stent	12	26.09	15.60–40.26
No percutaneous transluminal coronary angioplasty/stent	34	73.91	59.74–84.40
Patients on continuous veno-venous hemofiltration	16	34.78	22.68–49.23
Patients not on continuous veno-venous hemofiltration	30	65.22	50.77–77.32
Patients on extracorporeal membrane oxygenation	8	17.39	9.08–30.72
Patients not on extracorporeal membrane oxygenation	38	82.61	69.28–90.92
Patients on target temperature management	23	50.00	36.12–63.88
Patients not on target temperature management	23	50.00	36.12–63.88
Patients on target temperature management in <2 h	7	30.43	15.60–50.86
Patients on target temperature management in >2 h	16	69.57	49.14–84.40
Temperature ≥37.8 after 24 h^a^	20	47.62	33.36–62.28
Temperature <37.8 after 24 h^a^	22	52.38	37.72–66.64
Pupillary reflex + ^a^	24	57.14	42.20–70.88
Pupillary reflex –^a^	18	42.86	29.12–57.80
First evaluation of motor response ≤ M2	39	84.78	71.77–92.43
First evaluation of motor response > M2	7	15.22	7.57–28.23
Isoelectric electroencephalogram	10	21.74	12.26–35.57
Burst Suppression electroencephalogram	3	6.52	2.24–17.50
Low voltage electroencephalogram	4	8.70	3.44–20.33
Epileptiform electroencephalogram	8	17.39	9.08–30.72
Diffuse slowing electroencephalogram	17	36.96	24.53–51.40
Normal electroencephalogram	4	8.70	3.44–20.33
Discontinuous electroencephalogram patterns	17	36.96	24.53–51.40
Epileptiform electroencephalogram	8	17.39	9.08–30.72
Continuous electroencephalogram patterns	21	45.65	32.15–59.82
Electroencephalogram reactivity	10	21.74	12.26–35.57
Electroencephalogram unreactivity	36	78.26	64.43–78.26
Short-latency somatosensory evoked potentials +	29	63.04	48.60–75.47
Short-latency somatosensory evoked potentials −	17	36.96	24.53–51.40
10 mA Middle-latency somatosensory evoked potentials +	9	19.57	10.65–33.18
10 mA Middle-latency somatosensory evoked potentials −	37	80.43	66.82–89.35
50 mA Middle-latency somatosensory evoked potentials +	19	41.30	28.28–55.66
50 mA Middle-latency somatosensory evoked potentials −	27	58.70	44.34–71.72
50 mA Blood pressure reactivity +	37	80.43	66.82–89.35
50 mA Blood pressure reactivity –columns	9	19.57	10.65–33.18
Sedation during Neurophysiological recordings	22	52.38	37.72–40.26
Target temperature management during Neurophysiological recordings	12	26.09	15.60–40.26

^a^Missing data for four patients. *LUCAS* Lund University cardiac arrest system

## Results

### Survival outcome

**Fig. 1:**
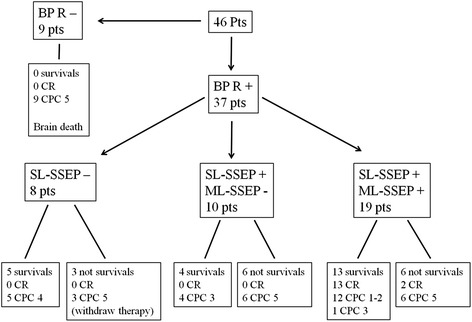
Distribution of patients on the outcome levels (survival, recovery of consciousness (CR) and cerebral performance category (CPC)) based on blood pressure reactivity (BP-R), short-latency somatosensory evoked potentials SL-SSEP and middle-latency SSEP (ML-SSEP). Of the patients, 6 with SL-SSEP and without 50 mA ML-SSEP died because of heart failure (n = 2), sepsis (n = 2) or bronchopneumonia (n = 2); 6 patients with SL-SSEP and 50 mA ML-SSEP died because of cardiogenic shock (n = 3), septic shock (n = 1), pulmonary hemorrhage (n = 1) or multiple organ failure (n = 1)

During hospitalization, 22 of 46 patients (48 %, 95 % CI 34.13–61.87 %) survived; all survivors had BP reactivity. Of those patients who did not survive, 9 (9.57 %, 95 % CI 10.65–33.18 %) had no BP reactivity and died within 7 days because of brain death; the other 15 patients (32.61 %, 95 % CI 20.87–47.03 %) died because of extra-neurological causes (Fig. 1).

Pearson’s chi-square test (corrected for multiple comparisons) attested that two variables were statistically significant to predict a fatal outcome at 6 months: pupillary reflex (*p* <0.005) and simplified EEG patterns (*p* <0.05), as shown in Tables 2 and 3. None of the results for analysis of the quantitative variables were statistically significant once the *p* values were corrected for multiple comparisons (*p* <0.05).

**Table 2:** Statistically significant variables

Variable	Significant outcome	*P* value	Sensitivity %	CI 95 %	Specificity %	CI 95 %	PPV %	CI 95 %	NPV %	CI 95 %
Pupillary reflex	Survival	<0.005	76.19	52.83–91.69	90.48	69.58–98.55	88.89	65.25–98.30	79.17	57.84–92.79
Continuous EEG patterns	Survival	<0.05	70.83	48.91–87.33	63.64	40.67–82.76	68.00	46.50–85.01	66.67	43.04–85.35
Epileptiform EEG pattern	Survival	<0.05	91.67	72.96–98.73	27.27	10.80–50.22	57.89	40.83–73.68	75.00	35.05–96.07
Discontinuous EEG patterns	Survival	<0.05	62.50	40.60–81.16	90.91	70.80–98.62	88.24	63.52–98.20	68.97	49.17–84.68
ML-SSEP at 50 Ma	Consciousness recovery	<0.001	87.10	70.15–96.29	100.00	78.03–100.00	100.00	87.11–100.00	78.95	54.43–93.82
SL-SSEP	Consciousness recovery	<0.05	54.84	36.04–72.67	100.00	78.03–100.00	100.00	80.33–100.00	51.72	32.54–70.54
ML-SSEP at 50 Ma	6 months CPC	<0.005	79.41	62.09–91.26	100.00	73.35–100.00	100.00	87.11–100.00	63.16	38.38–83.65

PPV positive predictive value, NPV negative predictive value, EEG electroencephalogram, ML-SSEP middle-latency somatosensory evoked potentials, SL-SSEP short-latency somatosensory evoked potentials, CPC cerebral performance category

**Table 3:** Significant parameters in the population categorized according to mortality, consciousness recovery and CPC at 6 months

Characteristic	Survivors			Non survivors			*P* value*
	Absolute frequency Number	Relative frequency %	CI 95 %	Absolute frequency number	Relative frequency %	CI 95 %	
Pupillary reflex + ^a^	19	45.24	31.23–60.05	5	11.90	5.19–24.99	<0.005
Pupillary reflex –^a^	2	4.76	1.31–15.79	16	38.10	25.00–53.19	
Discontinuous EEG patterns	2	4.35	1.20–14.54	15	32.61	20.87–47.03	<0.05
Epileptiform EEG	6	13.04	6.12–25.66	2	4.35	1.20–14.54	
Continuous EEG patterns	14	30.43	19.08–44.80	7	15.22	7.57–28.23	
Characteristic	Consciousness recovery			No consciousness recovery			*P* value*
	Absolute frequency number	Relative frequency %	CI 95 %	Absolute frequency number	Relative frequency %	CI 95 %	
SL-SSEP +	15	32.61	20.87–47.03	14	30.43	19.08–44.80	<0.05
SL-SSEP −	0	0.00	0.00–8.38	17	36.96	24.53–51.40	
50 mA ML-SSEP +	15	32.61	20.87–47.03	4	8.70	3.44–20.33	<0.001
50 mA ML-SSEP −	0	0.00	0.00–8.38	27	58.70	44.34–71.72	
Characteristic	CPC 1–2			CPC 3–5			*P* value*
	Absolute frequency number	Relative frequency %	CI 95 %	Absolute frequency number	Relative frequency %	CI 95 %	
50 mA ML-SSEP +	12	26.09	15.60–40.26	7	15.22	7.57–28.23	<0.005
50 mA ML-SSEP −	0	0.00	0.00–8.38	27	58.70	44.34–71.72	

*P value with Holm correction. ^a^Missing data for 4 patients. *EEG* electroencephalogram, *ML-SSEP* middle-latency somatosensory evoked potentials, *SL-SSEP* short-latency somatosensory evoked potentials, *CPC* cerebral performance category

**Fig. 2:**
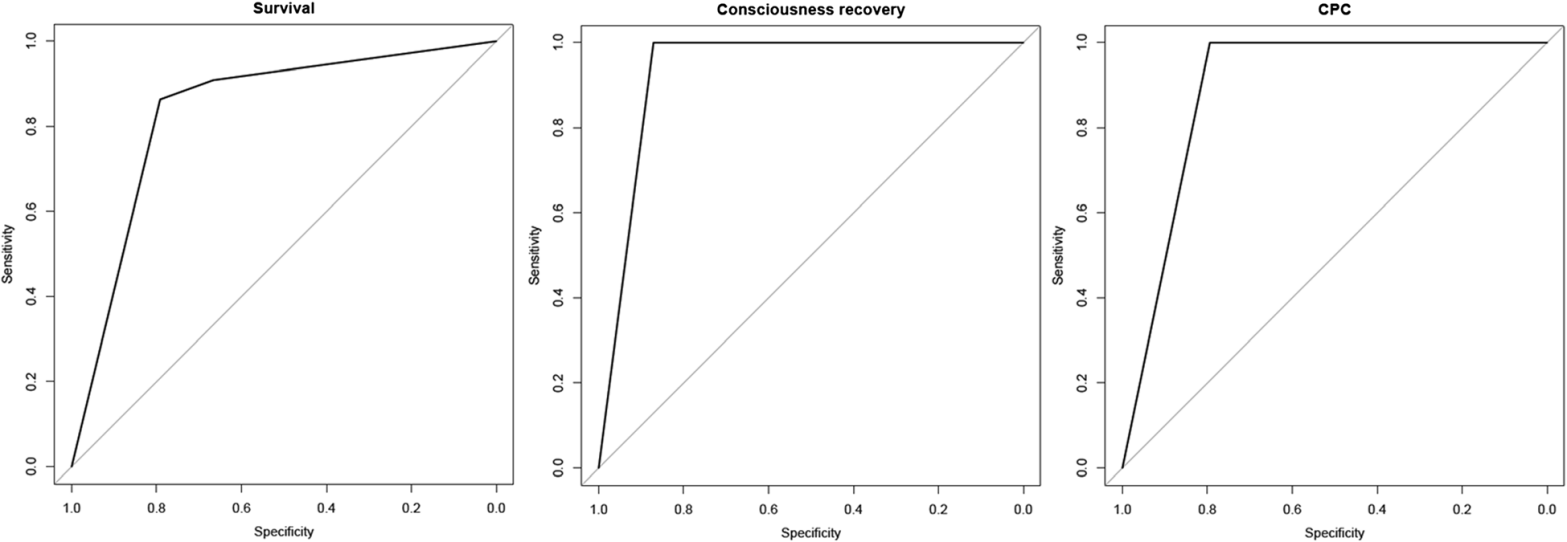
Receiver operator characteristic (ROC) curves for the three final logistic models. First panel (*left*): pupillary reflex ROC curve for survival; second panel (*middle*): 50 mA middle latency somatosensory evoked potentials (ML-SSEP) ROC curve for consciousness recovery; third panel (*right*): 50 mA ML-SSEP ROC curve for the 6-month cerebral performance category

The results of the logistic regression model show that the only relevant variable to predict a fatal outcome is the PR (odds ratio 23.399). The receiver operator characteristic (ROC) curve of such a model is plotted in the first panel of Fig. 2, and the area under the ROC curve (AUC) is 0.8371.

### Recovery of consciousness outcome

A total of 15 patients (32.61 %, 95 % CI 20.87–47.03 %) regained consciousness. All of them had SL-SSEP and 50 mA ML-SSEP; however, 2 of these 15 patients (13.33 %, 95 % CI 3.73–37.88 %) died during hospitalization because of non-neurological causes (Fig. 1), and 1 patient (6.67 %, 95 % CI 1.19–29.82 %) had CPC 3 at 6 months. Of the 31 patients (87.10 %, 95 % CI 71.15–94.87 %) who did not regain consciousness, 27 did not have 50 mA ML-SSEP; the other 4 patients (12.90 %, 95 % CI 5.13–28.85 %) died during hospitalization without recovery of consciousness because of non-neurological causes (Fig. 1).

Pearson’s chi-square test (after Holm’s correction for multiple comparisons) attested that two variables were statistically significant to predict recovery of consciousness: 50 mA ML-SSEP (*p* <0.001) and SL-SSEP (N20/P25; *p* <0.05), as shown in Tables 2 and 3. The results from the logistic regression model show that only 50 mA ML-SSEP was relevant in predicting a poor outcome (odds ratio 189.43). The ROC curve of such a model is plotted in the second panel of Fig. 2, and the AUC was 0.9355.

### CPC outcome

At 6 months, 12 of 46 patients (26.09 %, 95 % CI 15.60–40.26 %), that is, 12 of 13 (92.31 %, 95 % CI 66.69–98.63 %) patients who regained consciousness and survived, had a CPC of 1 or 2. All of them had SL-SSEP and 50 mA ML-SSEP. Of the 34 patients (79.41 %, 95 % CI 63.20–89.65 %) who had CPC 3–5, 27 did not have 50 mA ML-SSEP; 6 of these 34 patients (17.65 %, 95 % CI 8.35–33.52 %) had 50 mA ML-SSEP but had CPC 5 because they died during hospitalization (2 of them after recovery of consciousness); only 1 patient (2.94 %, 95 % CI 0.52–14.91 %) had 50 mA ML-SSEP but had CPC 3 at 6 months - this patient regained consciousness during hospitalization but ultimately evolved into a minimally conscious state (Fig. 1). No patient who was alive at ICU discharge died before the 6-month follow up.

Pearson’s chi-square test (after applying Holm’s correction for multiple comparisons) attested that only 50 mA ML-SSEP was statistically significant (*p* <0.005) in predicting CPC (Tables 2 and 3). The results from the logistic regression model show that only 50 mA ML-SSEP was relevant in predicting CPC (odds ratio 91.65). The ROC curve of such a model is plotted in the second panel of Fig. 2, and the AUC was 0.8971.

### Additional analysis

The time to the appearance of ML-SSEP was statistically lower than the time to the appearance of EEG reactivity (22 ± 12 vs. 196 ± 78 h, respectively, *p* <0.005) and was also lower than the time to the best Glasgow motor score (266 ± 304 h, *p* <0.001). Sedation and TTM did not influence the neurophysiological recordings. TTM did not prevent hyperthermia within 72 h.

### ML-SSEP distribution among the sample

Of the 19 patients who had ML-SSEP, 9 (47.37 %, 95 % CI 27.33–68.29 %) showed ML-SSEP at both 10 and 50 mA, and 10 (52.63 %, 95 % CI 31.71–72.67 %) had ML-SSEP only at 50 mA stimulation. The 9 patients who presented with 10 mA ML-SSEP had bilateral (n = 3), right (n = 3) and left (n = 3) ML-SSEP. The 10 patients who presented with only 50 mA ML-SSEP had bilateral (n = 7), right (n = 2) and left (n = 1) ML-SSEP. Pearson’ chi-square test attested that the presence of unilateral or bilateral ML-SSEP had no effect on predicting 6-month CPC.

## Discussion

This study suggests that the combination of a few neurophysiological and clinical parameters is useful to quickly stratify the prognosis of coma after CA (33 ± 22 h). In particular, PR and EEG patterns predict survival at 6 months, and the absence of BP reactivity seems to be related to brain death within 1 week. SL-SSEP and 50 mA ML-SSEP predicted consciousness recovery, and 50 mA ML-SSEP predicted CPC outcome at 6 months. The prognostic capability of 50 mA ML-SSEP occurred earlier than that of EEG reactivity. All of these parameters can be considered brain-damage-related indexes. Our data also showed that soon after CA the GCS has poor prognostic capability with respect to the neurophysiological parameters.

We find it more useful to stratify the level of good outcomes (in recovery of consciousness and CPC 1–2) at 6 months than to record only the best CPC score at any time within the first 6 months after CA [7], as recovery of consciousness reflects the end of coma caused by lower anoxic injury. As 50 ML-SSEP can quickly detect lower anoxic injury, it is important to focus therapies in these patients who can regain consciousness and eventually have a CPC of 1–2. In other words, having ML-SSEP does not necessarily imply a good outcome. A good outcome could be primarily related to the concomitant pathologies. Indeed, in our series, among those with 50 mA ML-SSEP, 7 patients did not have CPC of 1–2 at 6 months: 6 patients died during hospitalization (2 after recovery of consciousness, see Fig. 1), and 1 regained consciousness but had CPC 3 at 6 months - we suspect that this patient suffered from untreated status epilepticus, because the EEG reports during the rehabilitation time underlined a poor quality of recording due to many supposed muscular artifacts.

### Predictors of survival

Our overall mortality rate is lower than the rates from other recent series (48 %, vs. 70 % [44]), and is similar to the rate in the 1950s [45]; this difference can be explained by considering neurophysiological decisions to withdraw treatments in other trials [2, 6]. By contrast, in our hospital, an increased number of patients survived in a permanent vegetative state (PVS).

PR at ICU admission (recorded independently of TTM) seems to be the only parameter related to survival at 6 months, as the early absence of this brainstem reflex suggests a deep impairment of the central nervous system; however, this measure is less reliable than 72 h PR (10 % FPR vs. 0–4 %) [2, 3]. Interestingly, an absence of BP reactivity seems to constitute a marker of brain death within 1 week (specificity 100 %). These data are in line with Fugate’s [46] findings, in which the loss of cardiovascular regulation was related to brain death within one week. Moreover, patients with BP reactivity and absent PR who died after 1 week did so due to non-neurological causes. Interestingly, BP reactivity and PR seem to be related to the timing of death in our sample, probably because they are the expression of different levels of subcortical dysfunction, in the brain stem and the midbrain, respectively. We found, as shown in previous data [31], that BP reactivity was useful to stratify prognosis among patients with absent SL-SSEP into those who will die and those who could survive with a poor outcome (CPC 4).

### Predictors of consciousness recovery and CPC 1–2 at 6 months

Only SSEP evaluation was statistically significant in predicting recovery of consciousness and good CPC at 6 months. EEG was not statistically significant, but malignant EEG patterns had good specificity (93 % for consciousness and 100 % for CPC) in predicting a poor outcome. Most of the patients who regained consciousness (11 out of 15) and who had a good outcome (9 out of 12) had a continuous pattern, which is in line with the findings of Cloostermans et al. [7] and Rossetti [2]. Preservation of cortical synaptic transmission is the most critical factor in early recovery; however, specificity is not high (73 % for consciousness and 75 % for CPC), probably because of brain ischemia rather than TTM or sedation, based on early (33 h from CA) recordings [9, 10, 13, 15]. Indeed, our data showed that TTM (33–34 °C) and sedation did not change EEG and SSEP at all, reinforcing the fact that the greatest determinant of brain dysfunction both in intensive care [47] and in the operating room [48] is brain ischemia. Moreover, hypnotic sedation (e.g., midazolam and propofol) does not attenuate pain-related ML-SSEPs (like opioids do), as ML-SSEPs seem to be the neurophysiological expression of the activation of the brain-area network involved both in pain perception [11, 32] and other salient sensory input [33].

**Fig. 3:**
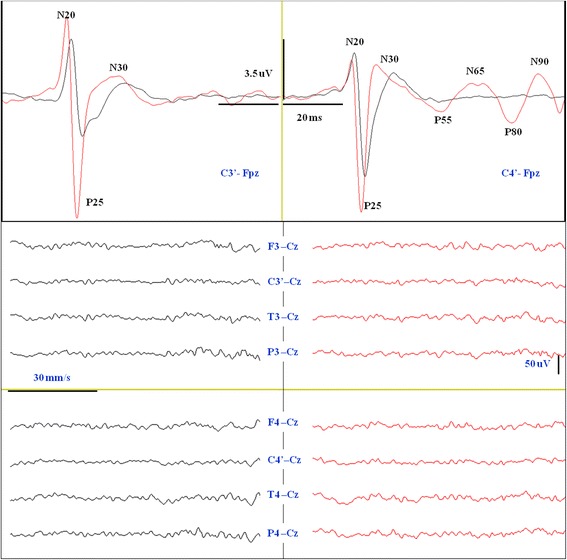
Somatosensory evoked potentials (SSEP) recording at 12 h after cardiac arrest in patient 5 of our series who had consciousness recovery and CPC 1–2. *Upper*: cortical SSEP (C3’/C4’–Fpz). Note the increase in amplitude and the decrease in latency of the N20/P25 evoked potentials at 50 mA electrical stimulation (*red line*) with respect to the baseline (*black line*) at 10 mA. Note also the appearance of ML-SSEPs (P55/N65 and P80/N90) at 50 mA stimulation on the left median nerve (*red line*). *Lower*: 8-channel electroencephalogram (EEG) (F3/F4-Cz, C3’/C4’–Cz, T3/T4–Cz, P3/P4–Cz) performed in the same session of SSEP. No significant background EEG pattern change is detected between baseline recordings (*black lines*) with respect to the stimulation at 50 mA (*red lines*)

We confirm the published data on SL-SSEP [49]: absent N20/P25 relates to lack of conscious recovery (100 % specificity) while present N20/P25 (SL-SSEP^+^) is not sensitive enough (55 % and 50 %) to predict a good neurological outcome. SSEP can explore both the primary somatosensory cortex (by SL-SSEP) and the secondary cortex (by ML-SSEP) in the range of 40–70 ms [17, 18]. ML-SSEP can be triggered by a 10 mA (9 patients) or 50 mA (19 patients) stimulus on median nerves. It is not unusual that ML-SSEP can also be activated by a low-intensity (10 mA) stimulus due to the salience of the electrical stimulus, thus assuming a lower extension of brain damage. However, 50 mA ML-SSEP has similar sensitivity (87 % vs. 90 % and 79 % vs. 88 %) but higher specificity (100 % vs. 40 % and 100 % vs. 42 %) than 10 mA ML-SSEP in predicting recovery of consciousness and CPC 1–2 at 6 months, respectively. Indeed, by stressing (at 50 mA) the somatosensory system and activating pain Aδ fibers, it is possible to explore the brain tissue that is still viable but not functioning, favoring the appearance of evoked cortical-cortical interactions (ML-SSEP) independent of the spontaneous cortical EEG reactivity, which is often suppressed by the brain ischemic penumbra (Fig. 3).

In our opinion, in the early phase of the postanoxic coma, the interpretation of SSEP reactivity seems to be simpler and more effective than that of EEG reactivity.

### Technical and procedural aspects

Muscle relaxant medication, which hides clinical evaluation, is a fundamental prerequisite to improve the reliability of neurophysiological evaluation by increasing the signal-to-noise ratio [31, 32], as greater noise might increase the numbers of false positives [1, 18]. In this study, false positives are minimized both by stimulation on the median nerve by the needle electrodes and by the earliness of the neurophysiological assessment (mean of 33 ± 22 h), which reduces the risk of technical difficulties related to possible limb edema or sepsis [50]. Moreover, the earliness of neurophysiological assessment prevents the possible interference of deep sedative medications like pentobarbital, which seems to suppress ML and long-latency SSEP in patients with severe head injuries [51]. However, experimental studies on recruitment of A delta fibers, as related to the strength of electrical stimulus applied on the peripheral nerve, show that pentobarbital does not suppress ML-SSEP [28, 52]. Our previous experience showed that, in anesthetized patients, propofol and midazolam did not blunt the cortical reactivity of electrical stimulation of A delta nerve fibers on the median nerves [11]. Moreover, it is important to underline that, even if several studies have shown that a stimulation rate above 1.5 Hz is associated with a decrease in ML-SSEP amplitude, the 50 mA stimulation compensates for the suboptimal recording paradigm, which consists of a 100 ms time base and a 3.3 Hz stimulation rate [53, 54].

Finally, we considered electrical stimuli on the median nerve sulcus to be a reproducible and noninvasive method to activate the A delta nerve fibers for triggering evoked and spontaneous brain reactivity; indeed this technique provides a way to evaluate EEG reactivity simultaneously with SSEP recording. Moreover the pain-related methodology is more sensitive to multimodal stimulations (somatosensory, auditory and visual), as these stimuli are under the threshold of salience in patients with consciousness disorders [55].

### Multimodal neurophysiological prediction of neurological outcome

**Fig. 4:**
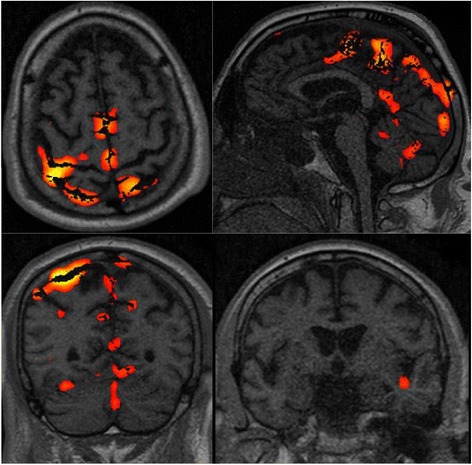
Functional magnetic resonance imaging (fMRI) (21 days after cardiac arrest (CA)) of patient 12 of our series who had a good outcome, showing the brain activation of the somatosensory, motor, premotor, left insula and cerebellum areas. Some artifacts are also visible. The neurophysiological recording performed at 24 h after CA showed pain-related somatosensory evoked potentials (ML-SSEP) and a diffuse, slowing, nonreactive electroencephalogram (EEG). The subsequent EEG patterns were characterized by epilectiform activity. This patient regained consciousness at 33 days after CA

From a neurophysiological point of view, simplified EEG patterns, SSEP (both SL and ML), PR, and BP reactivity constitute the key parameters that can quickly (within 72 h of CA) inform the prognostication of postanoxic coma because they are differently altered by ischemic injury with a cranio-caudal encephalic direction. Indeed, the finding of a normal or diffuse slowing EEG in the acute phase assumes the integrity of the brain structures that generate the spontaneous and evoked activity. While a benign reactive EEG pattern seems to be associated with 50 mA ML-SSEP (except for the presence of a neurological disease that alters the peripheral or central nerve transmission), 50 mA ML-SSEP seems to not always be associated with a benign reactive EEG pattern, as this pattern can appear later. The fact that EEG can be more suppressed than SSEP by hypoxic insult [56], hypothermia and anesthesia [11–15] shows that these neurophysiological examinations investigate different levels of brain function. Indeed, summed post-synaptic potentials (which generate EEG patterns) depend much more on the oxygen supply than do thalamus cortical cells (which generate cortical SL-SSEP) [56]. To supplement this work, our data showed that ML-SSEP triggered by painful stimulation at 50 mA occurs much earlier than a benign and reactive EEG does. In other words, the 50 mA ML-SSEPs stress the firing of the cortico-cortical interactions involved in pain perception (in the insula, SII, and cingulate cortex) independently of the spontaneous cortical activity hidden by the ischemic penumbra. In this way ML-SSEP seems to represent a higher-order complexity of the somatosensory afferent pathway sensing mode, and the recovery of normal spontaneous synaptic functions seems to be critical in the recovery of the sending mode through the efferent behavioral pathway. The reliability of 50 mA ML-SSEP in predicting preserved cortical connections is also confirmed by fMRI neuroimaging data. Patients who regained consciousness and had a good outcome showed the activation of brain areas involved in the detection of pain [32], as shown in Fig. 4, which represents the twelfth patient of our series.

Interestingly, the early absence of ML-SSEP after CA is a marker of EEG synaptic depletion associated with insufficient recovery of motor-behavioral function and an evolution towards a minimally conscious state [31, 32].

### Limitations

This study has some important limitations. First, the neurophysiologist reading the ML-SSEPs (and EEG) was not blinded to the patients’ clinical status and outcomes. A multicenter prospective study should be carried out in order to share and validate this methodology and fully estimate the effectiveness of 50 mA ML-SSEP in predicting neurological outcome in postanoxic patients. Further analysis should also consider ML-SSEP amplitude and laterality to better define long-term outcome and cognitive status while using a more detailed scale than CPC (e.g., Level of Cognitive Function, Coma Recovery Scale-Revised or neuropsychological evaluation). Moreover, we believe that the pain-related SSEP method could have false positive results if the patient suffers from A delta fibers disease. Finally, the impact of rehabilitation treatments on the final outcome of the patients should be evaluated.

## Conclusions

This study shows that the combination of BP reactivity, PR, simplified EEG, SL-SSEP, and ML-SSEP constitute the key parameters that can quickly (within 72 h of CA) inform the prognostication of coma after CA. The Glasgow neurophysiological analog, obtained through the activation of ML cortical potentials with painful electrical stimulation on the median nerves, seems to detect the residual higher-order brain processing function. This predicts a good neurological prognosis earlier than EEG reactivity because this last one reflects the spontaneous electrical activity of brain areas that can be stunned by brain ischemia. Conversely, the absence of ML-SSEPs seems to be associated with a bad outcome. According to our opinion this method should be integrated into routine neurophysiological evaluation and used to funnel ICU treatments to patients with a good chance of recovery.

## Key messages

- EEG, pupillary reflex, short- and middle-latency SSEP, and blood pressure reactivity constitute key parameters that can quickly (within 72 h of CA) inform the prognostication of postanoxic coma
- Middle-latency pain-related SSEPs detected early after a CA are associated with a good neurological prognosis in the absence of concomitant extra-neurological pathologies
- The prognostic capability of pain-related middle-latency SSEP was demonstrated to occur earlier than that of EEG reactivity

## Abbreviations

AS: asystole
AUC: area under the curve
BPR: blood pressure reactivity
CA: cardiac arrest
CAG: coronary angiography
CPC: cerebral performance category
CPR: cardiopulmonary resuscitation
CVVH: continuous veno-venous hemofiltration
ECMO: extracorporeal membrane oxygenation
EEG: electroencephalogram
EMR: evaluation of motor response
fMRI: functional magnetic resonance imaging
GCS: Glasgow coma scale
IABP: intra-aortic balloon pump
ICU: intensive care unit
IHCA: in-hospital cardiac arrest
LUCAS: Lund University cardiac arrest system
ML-SSEP: middle-latency somatosensory evoked potentials
OHCA: out-of-hospital cardiac arrest
PaCO_2_: partial pressure of arterial carbon dioxide
PaO2: partial pressure of arterial oxygen
PEA: pulseless electrical activity
PIDS: patient’s intravascular drug support
PR: pupillary reflex
PTCA: percutaneous transluminal coronary angioplasty
ROC: receiver operator characteristic
ROSC: return of spontaneous circulation
SII: secondary somatosensory area
SL-SSEP: short-latency somatosensory evoked potentials
TTM: target temperature management
VF: ventricular fibrillation
VT: ventricular tachycardia
